# Osteopontin Expression during Early Cerebral Ischemia-Reperfusion in Rats: Enhanced Expression in the Right Cortex Is Suppressed by Acetaminophen

**DOI:** 10.1371/journal.pone.0014568

**Published:** 2011-01-21

**Authors:** Sunanda S. Baliga, Gary F. Merrill, Mari L. Shinohara, David T. Denhardt

**Affiliations:** 1 Department of Cell Biology and Neuroscience, Rutgers University, Piscataway, New Jersey, United States of America; 2 Department of Immunology, Duke University Medical Center, Durham, North Carolina, United States of America; National Institutes of Health, United States of America

## Abstract

Osteopontin (OPN) is a pleiotropic protein implicated in various inflammatory responses including ischemia-reperfusion (I-R) injury. Two distinct forms of the protein have been identified: an extensively studied secreted form (sOPN) and a less-well-known intracellular form (iOPN). Studies have shown that increased OPN expression parallels the time course of macrophage infiltration into injured tissue, a late event in the development of cerebral infarcts. sOPN has been suggested to promote remodeling of the extracellular matrix in the brain; the function of iOPN may be to facilitate certain signal transduction processes. Here, we studied OPN expression in adult male Sprague-Dawley rats subjected to global forebrain I-R injury. We found iOPN in the cytoplasm of both cortices and the hippocampus, but unexpectedly only the right cortex exhibited a marked increase in the iOPN level after 45 min of reperfusion. Acetaminophen, a drug recently shown to decrease apoptotic incidence, caspase-9 activation, and mitochondrial dysfunction during global I-R, significantly inhibited the increase in iOPN protein in the right cortex, suggesting a role for iOPN in the response to I-R injury in the right cortex.

## Introduction

Osteopontin (OPN) is engaged in a variety of cellular processes ranging from bone resorption to immune cell activation, remodeling of the extracellular matrix and inhibition of apoptosis [1], [2], [3]. OPN protein levels are elevated in the days following cerebral I-R [4], [5], [6]. OPN expression parallels the time course of macrophage infiltration into the infarct [5], a late event in the development of cerebral infarcts [7]. This suggests that the upregulation of OPN is delayed until brain matrix remodeling is underway [5]. While delayed expression of OPN in stroke has been reported, there are little data on its role in cerebral injury early during cerebral I-R.

OPN can exist in two forms: secreted (sOPN) and intracellular (iOPN). sOPN can engage various receptors (certain integrins and CD44 variants) on the cell surface, stimulating signal transduction pathways and cell adhesion [8], [9]. Certain of these receptors are upregulated following transient global cerebral I-R [10]. Extensive post-translational modifications (glycosylation, phosphorylation) can modify the interaction of OPN with other proteins. OPN can be cleaved by thrombin, exposing a cryptic attachment motif that is capable of engaging additional integrins [11]. iOPN, which lacks the signal sequence that targets the protein to secretory vesicles, possibly due to a down-stream alternative translational initiation signal, is expressed in dendritic cells and macrophages of the immune system [12]. In patients with advanced stages of Alzheimer's disease, iOPN levels are increased in pyramidal neurons compared to normal human brain [13]; the authors suggested that iOPN may play a role in cell cycle progression, neuronal remyelination, and/or the formation of protein aggregates in Alzheimer's Disease. The role of iOPN in the cellular response to stroke has not been studied.

Here, we evaluated OPN expression early after cerebral I-R in three different areas of the brain. Next, we determined if the form of OPN we detected in cortical brain tissue was secreted or intracellular. Lastly, we investigated OPN expression in the presence of acetaminophen (ACET), a drug recently shown to reduce apoptosis and mitochondrial dysfunction in early cerebral I-R [14].

## Methods

All chemicals were purchased from Sigma Aldrich, Inc. (St. Louis, MO). The anti-OPN antibody 2A1 was developed and characterized in our laboratory [15]. Other antibodies used were obtained from Santa Cruz Biotech, Santa Cruz, CA unless otherwise indicated.

### Animals

Rats weighing 350–400 g were obtained from Ace Animals, Inc (Boyertown, PA) and housed in AAALAC- accredited facilities at Rutgers University. Four animals were used in each treatment group described below. All animal housing conditions, surgical protocols and postoperative care were reviewed and approved by the Rutgers University Institutional Animal Care and Use Committee and were carried out in accordance with the *National Institute of Health Guide for the Care and Use of Laboratory Animals* (NIH Publications No. 80-23; revised 1996).

### Surgical procedure (2VO/HYP)

Transient global forebrain ischemia was induced using the two-vessel occlusion and hypovolemic hypotension (2VO/HYP) model [16]. The carotid arteries supply blood to a significant portion of the brain. If carotid artery blood flow is compromised, pressure in the vertebral arteries increases in compensation [17]. In this model, blood flow in the vertebral arteries is reduced by withdrawing blood from the femoral artery and then initiating ischemia by clamping both carotid arteries. This procedure causes significant damage to the hippocampus and cortex after 15 min of ischemia [16], [18]. Additionally, surgical manipulation is minimized and reperfusion is readily accomplished.

**Figure 1:**
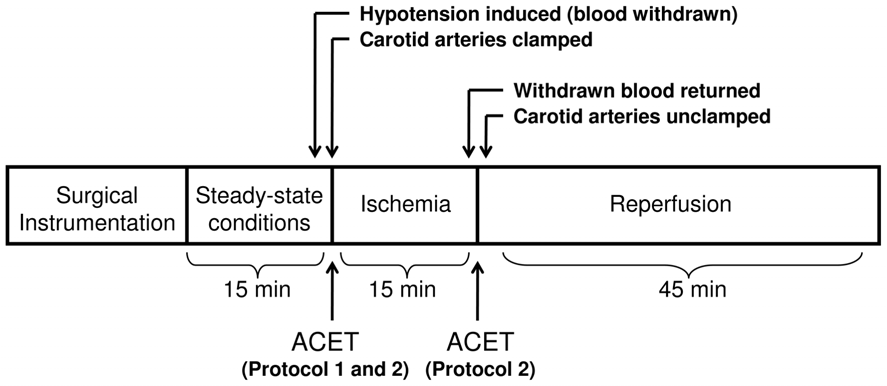
Schematic of experimental timeline. Rats were surgically instrumented and maintained under steady-state conditions for 15 min prior to initiating I-R. At that point, animals were rendered hypotensive (mean arterial pressure ∼55 mm Hg) by withdrawing 5 mL of blood, and the carotid arteries were clamped to induce ischemia. Reperfusion was begun by unclamping of the carotid arteries and returning the withdrawn blood to the animal. Control animals were surgically instrumented but were not subject to hypotension or I-R. In Protocol 1, acetaminophen (ACET) (15 mg/kg) was only administered prior to ischemia, whereas in Protocol 2, ACET (15 mg/kg) was administered prior to ischemia and again immediately before the onset of reperfusion.

Rats were anesthetized with an intraperitoneal (i.p.) injection of ketamine:xylazine (80∶12 mg/kg) with additional i.p. doses of ketamine (80 mg/kg) administered throughout the experiment as necessary. A ventral midline incision was made and the right jugular vein cannulated. The carotid arteries were isolated and marked with suture. The femoral arteries were cannulated to monitor arterial blood pressure and to extract blood from the animal. Lastly, the animal was intubated and connected to a small animal respirator (Model 683, Harvard Apparatus, Holliston, MA) to maintain physiological blood pH. The animals maintained under these conditions for an additional 60 min are referred to as the controls (see figure 1 below). Heparin (250 U/kg) was administered to inhibit blood clotting and hypotension (∼55 mm Hg) was induced by withdrawing blood via the femoral artery prior to ischemia. Withdrawn blood was placed in a heparinized vial and maintained at 37°C. Ischemia was initiated by clamping the carotid arteries for 15 min. Reperfusion was initiated by unclamping the carotid arteries and returning withdrawn blood to the animal. ACET was administered via the jugular vein. Body temperature was maintained within physiological range. The animal was sacrificed after 45 min of reperfusion.

### Treatment protocols (Figure 1)

#### Controls

Rats were instrumented as described above but were not subjected to I-R. They were maintained under steady-state conditions for 75 min.

#### Protocol 1 Vehicle (P1VEH)

Rats were instrumented and subjected to hypotension, carotid artery occlusion and 60 min of I-R. Vehicle (0.2–0.3 mL of 0.15 M saline) was administered immediately prior to ischemia.

#### Protocol 1 ACET (P1ACET)

Rats were instrumented as above and subjected to hypotension, carotid artery occlusion and I-R. A 15 mg/kg dose of ACET (0.125 M in 0.15 M saline, 0.2–0.3 mL) was administered immediately prior to ischemia.

#### Protocol 2 Vehicle (P2VEH)

Rats were instrumented as above and subjected to hypotension, carotid artery occlusion and I-R. A 0.2–0.3 mL dose of 0.15 M saline was administered immediately prior to ischemia and again immediately prior to reperfusion. The doses were administered separately to avoid a large infusion volume, which is known to cause vascular damage [19].

#### Protocol 2 ACET (P2ACET)

Rats were instrumented as above and subjected to hypotension, carotid artery occlusion and I-R. A 15 mg/kg dose of ACET (0.125 M in 0.15 M saline, 0.2–0.3 mL) was administered immediately prior to ischemia and again immediately prior to reperfusion.

At the end of reperfusion, animals from all groups were sacrificed by transcardial perfusion of ice-cold PBS until the blood was completely flushed out. For immunocytochemical analysis, animals were also transcardially perfused with 4% paraformaldehyde in PBS (PFA). In P1VEH and P1ACET rats, brains were extracted and the left and right cerebral cortices as well as the hippocampus were evaluated. In P2VEH and P2ACET rats, only the right cortex was evaluated. Tissues used for cytosolic/mitochondrial fractionation and Western blotting were obtained from animals transcardially perfused with ice-cold PBS.

### Evaluation of OPN expression in brain tissue

The left and right cortical hemispheres from control, P1VEH and P1ACET rats were placed in 4% PFA overnight at 4°C. Tissue was cryoprotected by consecutive 8-h incubations in 10, 20 and 30% sucrose respectively at 4°C. Tissue was prepared for cryosectioning by incubation in 30% sucrose/50% OCT (v/v) for 1 h followed by incubation in 100% OCT at 4°C for 1 h. The tissue was then embedded in OCT over dry ice and 10 µm-thick sections were obtained using a cryostat (model L-CM1900, Leica, St. Gallen, Switzerland). Slides were stored at −70°C prior to analysis. Sixteen slides (four slides per animal) were analyzed from each treatment group.

For immunocytochemical analysis, sections were first washed in PBS. Endogenous hydrogen peroxidases were quenched using 0.3% H_2_O_2_ in PBS for 30 min. Sections were then blocked in 1% BSA for 1 h followed by incubation with 2A1 (0.02 µg/µL) overnight at 4°C in a humidified chamber. The 2A1 antibody used in this study was generated in our laboratory; it binds to an epitope [*(A/V)IPVAQ*] centrally located in the C-terminal thrombin cleavage fragment about 50 amino acids downstream of the RGD integrin-binding site (humans, mice, rats, rabbits; not cows, pigs or chickens). The epitope is not subject to post-translational modifications that can interfere with antibody-epitope recognition [15]. A few sections were used as negative controls, where the primary antibody was omitted. Slides were then incubated with secondary HRP-conjugated goat anti-mouse antibody (1∶50, BioRad, Hercules, CA) for 1 h. Immunostaining was developed by the peroxidase-antiperoxidase procedure using 3-3′-diaminobenzidine (Dako, Glostrup, Denmark) as the cosubstrate. Sections were counterstained with hematoxylin before mounting. Four 20X fields were chosen to best reflect the overall immunostaining of the cerebral cortex of each slide. Cytoplasmic expression of iOPN was determined as described [13]. OPN abundance was quantified using Adobe Photoshop Software [20]. Four 20X fields were digitally acquired from each cortical tissue section and opened using Photoshop software. The mean values were normalized to background values and compared between the control, P1VEH and P1ACET groups. Staining intensities are expressed as a percentage of control values.

### Evaluation of injury from ischemia-reperfusion

A key characteristic of damaged neurons is the presence of pyknotic (condensed) nuclei. Morphological changes in the CA1 region of the hippocampus were determined as described [21]. Tissue sections from the left and right hippocampi of control and P1VEH brains were stained with hematoxylin and eosin. Neurons exhibiting pyknotic nuclei were counted (magnification, 20X). Pyknotic neuronal cell counts were compared between control and P1VEH brains for left and right hippocampi respectively.

### Cytosolic/mitochondrial fractionation

Tissue samples were separated into cytosolic and mitochondrial fractions as previously described [14]. Each dissected brain region was placed in ice-cold homogenization buffer (210 mM mannitol, 7 mM sucrose, and 5 mM 4-morpholinopropanesulfonic acid, pH 7.4, 25°C, with 1 mM PMSF and protease inhibitor cocktail) and homogenized with 10–15 strokes using a Dounce Homogenizer (Fisher Scientific, Waltham, MA). Samples were centrifuged at 4000 *g* for 10 min at 4°C to remove intact cells and cellular debris. The supernatant was transferred to a separate tube and centrifuged again at 7000 *g* for 10 min at 4°C. The supernatant (the cytosolic fraction) was carefully removed from the pellet (the mitochondrial fraction).

### Detection of OPN and cytochrome c content by Western Blotting

The protein concentration in each sample was determined using the Bradford method. Cytosolic and mitochondrial proteins (10 µg) were resolved on a 12% SDS-polyacrylamide gel at 100 V for 2 h. Proteins were then transferred to an Immobilon-P membrane (Millipore, Billerica, MA) and blocked with 2% BSA followed by incubation with primary mouse monoclonal antibody (2A1, 0.5 ng/mL) overnight at 4°C. Membranes were washed with TBS-T buffer and then incubated with HRP-conjugated goat anti-mouse secondary antibody (1∶6000, Bio-Rad, Hercules, CA) for 45 min. Blots were developed using the ECL^+^ reagent (Amersham Biosciences, Piscataway, NJ). Film was analyzed using NIH-developed software (Image J, http://rsbweb.nih.gov/ij/). Membranes were stripped using mild stripping buffer (0.2 mM glycine, 0.003 mM SDS, 0.01% Tween-20, pH 2.2) and blocked with 2% BSA for 2 h at room temperature. Then, membranes were incubated with polyclonal rabbit anti β-tubulin antibody (1∶1000) and secondary HRP-conjugated goat anti-rabbit antibody (1∶4000, Bio-Rad, Hercules, CA). Blots were developed and analyzed as above; protein molecular weight markers were the Benchmark Pre-stained Protein Ladder (Invitrogen, Carlsbad, CA). The level of OPN in each cytosolic sample was expressed as a ratio to β-tubulin. Each experiment was performed four times with consistent results.

Mitochondrial samples were probed with anti-cytochrome c antibody to determine the differences in cytochrome c content between groups. Membranes were stripped and incubated with a primary polyclonal goat antibody against mitochondrial-specific, voltage-dependent anion channel protein (VDAC, 1∶1000) and then incubated with secondary donkey HRP-conjugated anti-goat antibody (1∶6000). Although the mitochondrial number of each sample was not determined, equal amounts of mitochondrial protein from each sample were analyzed to ensure objective comparison between treatment groups. Blots were obtained as described above. All experiments were performed in duplicate, yielding comparable results.

### Statistical analysis

Data are presented as means ± SD. Statistical differences between two groups were assessed using two-tailed, unpaired Student's *t* tests. Differences among three groups were analyzed using one-way analysis of variance (ANOVA) followed by Tukey's post-test for means comparison (GraphPad Software, La Jolla, CA). Significance was accepted at P<0.05.

## Results

### iOPN expression in the cytoplasm of the cortex: enhanced by ischemia-reperfusion only in the right cortex

**Figure 2:**
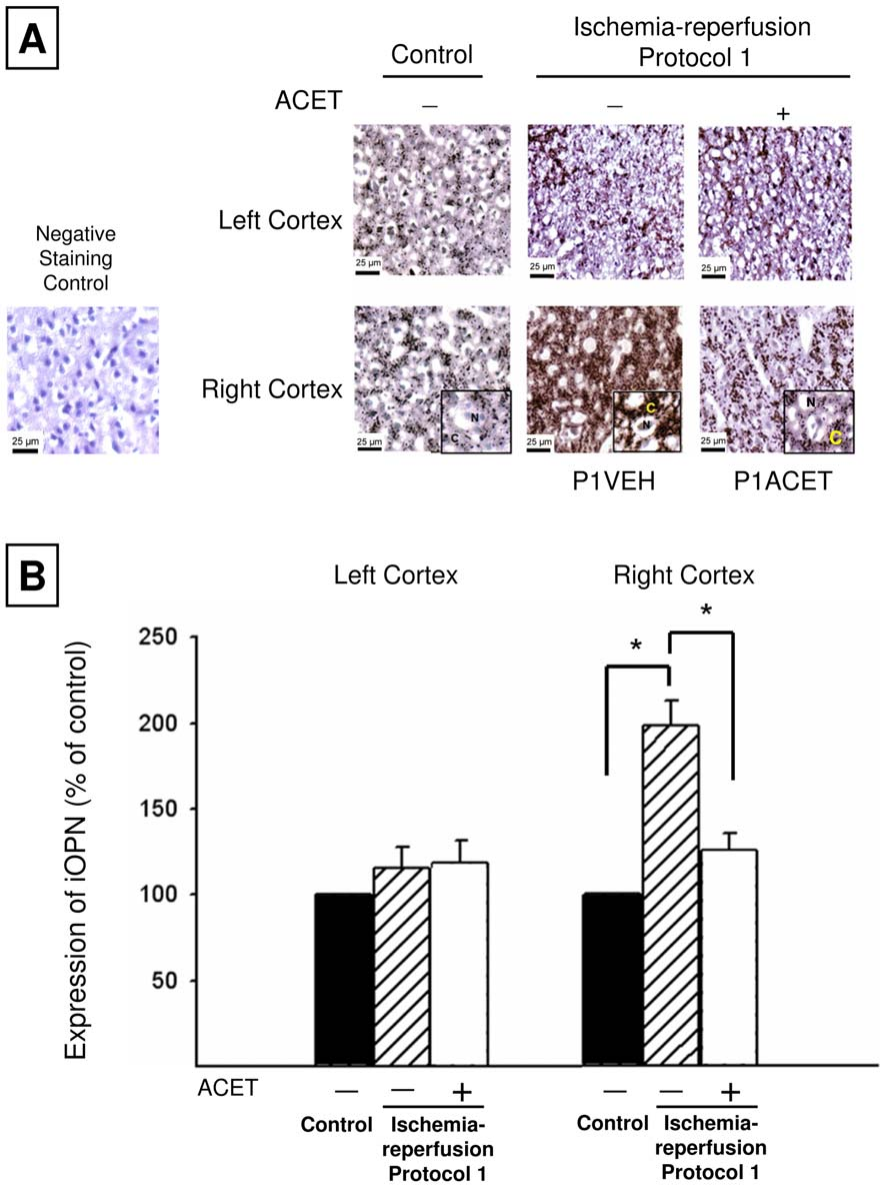
Immunocytochemical analysis of iOPN expression. **A**: Representative sections exhibiting iOPN expression from the left (top) and right (bottom) cortex in control, P1VEH and P1ACET animals. We detected iOPN in the cytoplasm (labeled C, insets). A negative staining control from the right cortex is shown for comparison. In the right cortex, higher iOPN expression was evident in vehicle rats compared to control and ACET (15 mg/kg)-treated rats. **B**. The intensity of staining was quantified as described in Methods. The right cortex of vehicle rats shows a significant increase in iOPN expression compared to ACET (15 mg/kg)-treated rats. *P<0.05 determined using one-way ANOVA followed by Tukey's post test. Error bars indicate S.D. Scale bars  = 25 µm.

We employed immunocytochemistry on tissue sections obtained from the left and right hemispheres to investigate the presence of OPN and its response to I-R. We observed a staining pattern in brain tissue (Figure 2A and insert) consistent with a cytoplasmic localization iOPN. The inserts show more magnified views, confirming the presence of iOPN in the cytosol. Because the brain was flushed with PBS prior to preparation for immunocytochemistry, it would be essentially free of blood cells or plasma that might contain sOPN. Interestingly, iOPN protein was uniquely elevated in the right cortex of the P1VEH animals following I-R injury, and ACET inhibited that increase (Figure 2A, B). Please note that VEH designates animals that received only the vehicle in which the acetaminophen is dissolved.

### Response to I-R injury in the right and left hippocampi

**Figure 3:**
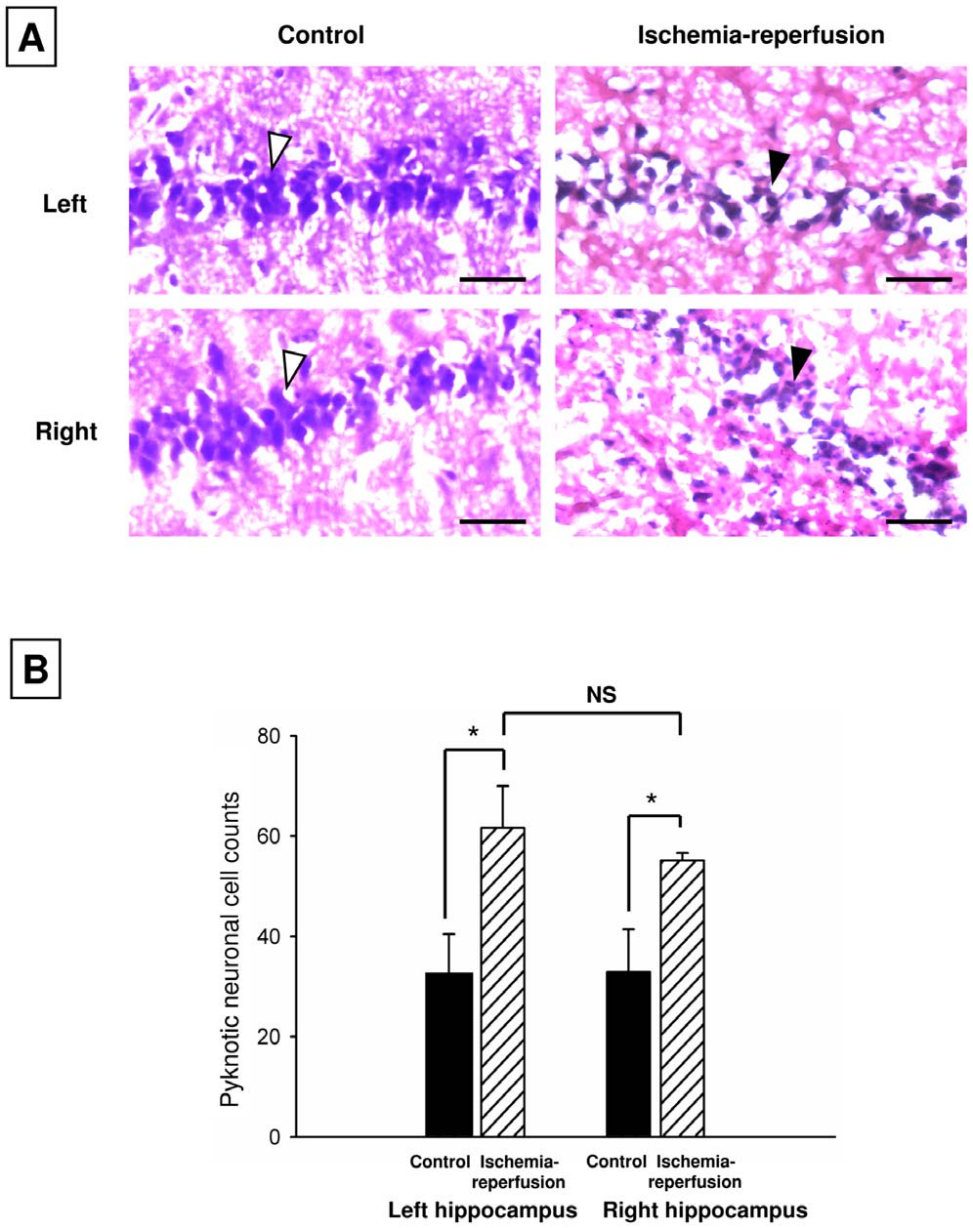
Neuronal damage does not differ between left and right hemispheres in the CA1 region of the hippocampus. **A**. Representative hematoxylin and eosin-stained tissue sections show pyknotic neurons (black arrowheads) in the CA1 regions of the hippocampi obtained from controls and P1VEH rats. Sections of the CA1 regions of the left and right hippocampi regions from control rats show an abundance of normal neurons (white arrowheads). **B**. Quantitative analysis reveals a significantly higher number of pyknotic neurons in vehicle rats compared to corresponding control animals. There are no differences in pyknotic neuronal cell count between left and right hippocampi regions of P1VEH rats. Scale bars  = 10 µm. Error bars indicate S.D. *P<0.05 determined using Student's *t* test.

Analysis of tissue from the CA1 regions of the hippocampi from the left and right hemispheres (Figure 3A) revealed a significant increase in pyknotic neurons in the P1VEH group compared to the control, for both left and right hippocampi, indicating that there was more neuronal damage in the P1VEH group than in the instrumented control rats. The nuclei in hippocampal sections obtained from control animals are larger and stain more darkly than the condensed nuclei in the sections from vehicle animals. There were no significant differences in pyknotic neuronal counts between left and right hippocampi in the P1VEH group, suggesting that the extent of neuronal damage in the P1VEH group was similar between left and right hippocampi (Figure 3).

### OPN expression in the hippocampus

**Figure 4:**
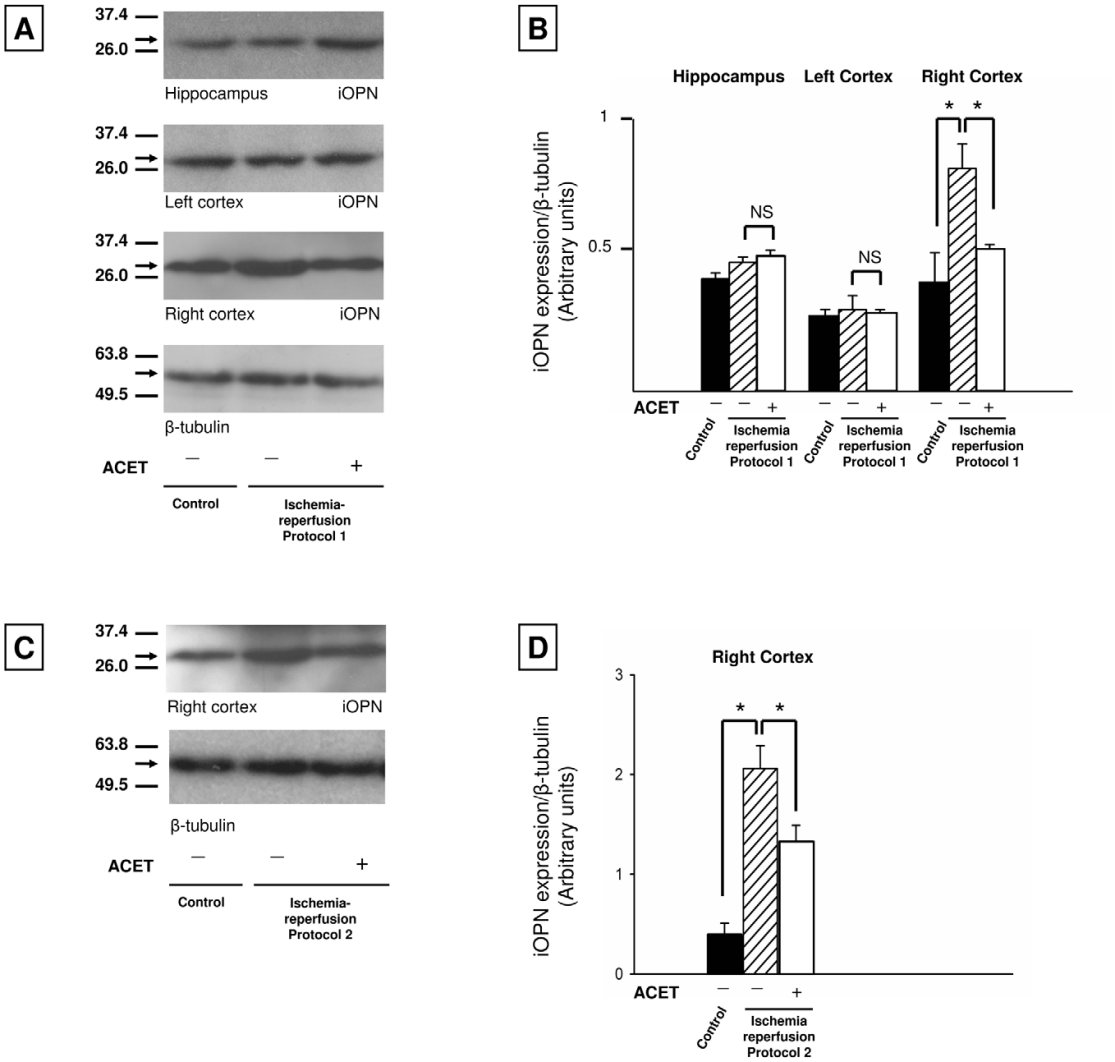
iOPN expression in the hippocampus, left cortex and right cortex. **A**. Representative Western blots of cytosolic fractions from the hippocampus, left cortex and right cortex of control, P1VEH, and P1ACET rats reveal a ∼30 kDa OPN species; species of lower mobility were not detected. Control animals were instrumented, but not subject to I-R. **B**. Quantitative densitometric analysis of iOPN bands normalized to their corresponding β-tubulin bands confirmed a significant increase of iOPN in vehicle rats in the right cortex. **C**. Representative Western blots of cytosolic fractions from the right cortex of control, P2VEH and P2ACET rats. **D**. Quantitative densitometric analysis of iOPN bands normalized to their corresponding β-tubulin bands confirmed a significant increase in iOPN expression in P2VEH rats that is reduced by about 50% in the P2ACET rats. Error bars indicate S.D. *P<0.05 determined using one-way ANOVA followed by Tukey's post test.

The hippocampus is one of the regions in the brain severely affected by the 2VO/HYP procedure used in this study [16], thus we chose to examine this region. Western blot analysis of the cytosolic fractions of hippocampus tissue revealed a 30-kDa species (Figure 4A) as shown by others [4]; OPN species in the 55–70-kDa range (full-length and post-translationally modified) were not detected.

### iOPN is highly expressed in the right cortex during early I-R: Western blot analysis

Cerebral lateralization (biochemical, physiological, functional differences between right and left halves of the brain) is a poorly understood phenomenon, which we discuss further below. These differences can cause the two halves of the brain to respond differently to the same stimulus, I-R injury for example. In the Western blot analysis of cytosolic fractions from the left and right cortex of control, P1VEH and P1ACET rats revealed a 30-kDa band (Figure 4A). OPN species in the 55–70-kDa region were again not detected. The right cortex of group P1VEH exhibited a marked increase in iOPN expression compared to the other groups (Figure 4B), consistent with the results presented above in figure 2B. To confirm our results with protocol 1, we assayed iOPN expression in the right cortex of P2VEH and P2ACET rats (Figure 4C). We observed a similar pattern where iOPN expression was markedly increased in P2VEH rats compared to control and P2ACET animals (Figure 4C).

### ACET prevents mitochondrial release of cytochrome c

**Figure 5:**
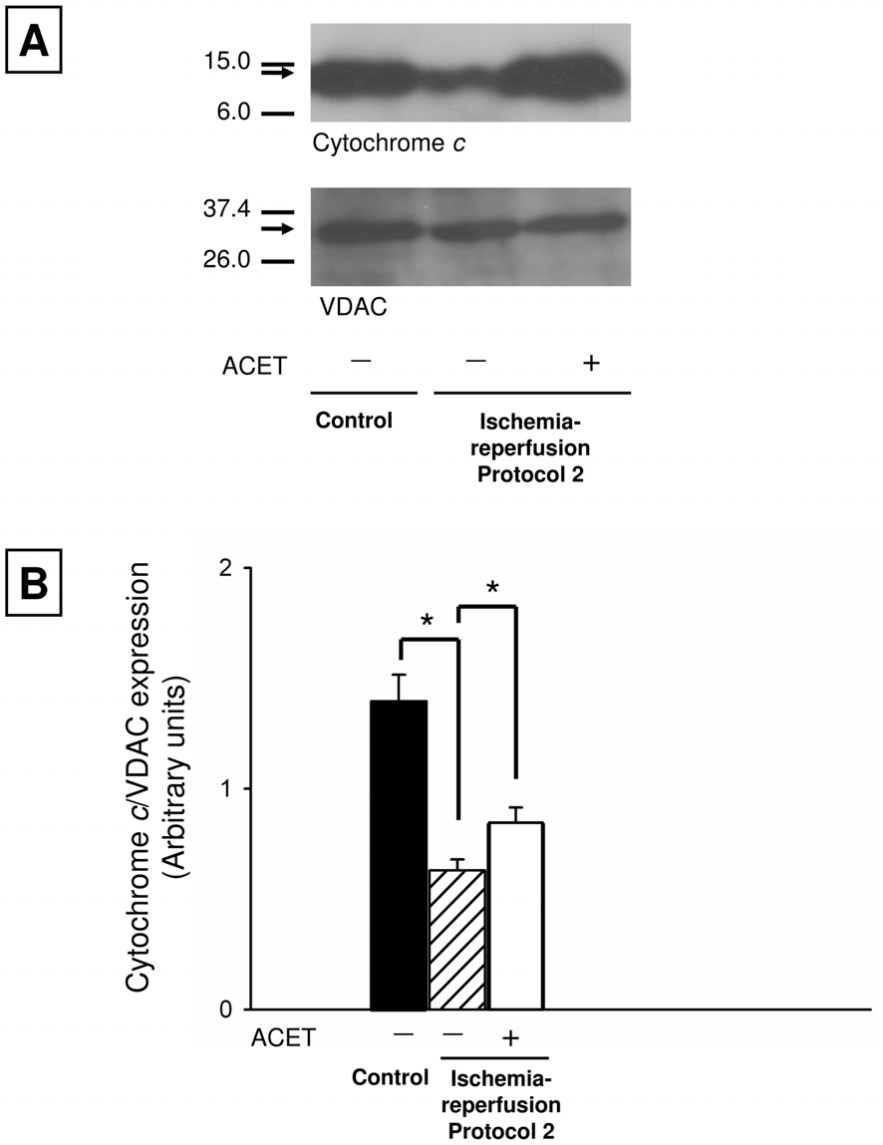
Acetaminophen (ACET) maintains mitochondrial cytochrome c content in rats subjected to Protocol 2. **A**: Representative Western blots of the mitochondrial fractions from the right cortex of control, P2VEH, and P2ACET rats. **B**: Quantitative densitometric analysis of cytochrome c bands normalized to their corresponding VDAC bands confirms a significant decrease in cytochrome c content in vehicle rats. Error bars indicate S.D. *P<0.001 determined using Student's *t* test.

We previously showed that ACET treatment (15 mg/kg) mitigated the extent of mitochondrial disruption resulting from I-R injury similar to that induced here [14]. Cytochrome c, which is normally sequestered in the mitochondria, triggers apoptosis when released into the cytosol [22]. We employed Western blotting to determine whether the ACET treatment in Protocol 2 was effective in preventing release of cytochrome c. Our data (Figure 5) show that mitochondrial cytochrome c content was maintained with ACET treatment, whereas P2VEH rats exhibited a marked decrease in cytochrome c content.

## Discussion

### iOPN expression in the brain

Here, we report for the first time that iOPN protein levels are specifically increased in the right cortex early after transient global cerebral ischemia/reperfusion (15/45 min) in rats, suggesting a role for the protein as a responder to stroke-induced cell damage. Up-regulation, which was partially suppressed by ACET, was evident in the right cortex but not the hippocampus (right or left) or left cortex. Both common carotid arteries were occluded in the 2VO/HYP model [16] used in this study; thus experimental manipulation is unlikely to contribute to the differences in cortical expression of iOPN.

Little is known about the expression patterns and role of iOPN early during cerebral I-R. Changes in gene expression can occur as early as 15 min following a cerebral insult [23] and protein synthesis is significantly affected as well [24]. Cerebral ischemia can initiate the transcription of immediate early genes – some required for cellular recovery– that code for transcription factors [25], while inhibiting translation [22]. Increased transcription, translation, or stability of the mRNA and/or protein could account for the increased abundance of iOPN after I-R injury. Differences in spatio-temporal up-regulation of OPN mRNA have been described previously in the hippocampus and the striatum following global forebrain ischemia [26].

Baliga et al. [14] recently demonstrated that a single dose of ACET (15 mg/kg) reduced cerebral injury, apoptosis-mediated DNA fragmentation, and mitochondrial release of cytochrome c following early cerebral I-R. Here, we administered an additional dose (15 mg/kg) at the end of ischemia and found a reduction in iOPN expression similar to that noted with a single dose. We found that the administration of two doses had a similar action to the single dose, causing a similar reduction in mitochondrial release of –cytochrome *c* (Figure 5).

### Cerebral lateralization: the underlying biochemistry

Gene expression in the left and right cortices varies during development [27] and in response to certain physiological or pathological inputs [28]. Clinical studies have shown that the cortical location of the lesion (left vs. right) in stroke patients leads to varied outcomes regarding speech, motor and memory rehabilitation [29], [30]. In our study, no significant differences in iOPN protein levels were found between the left and right cortical tissue obtained from control animals. Unexpectedly however, in response to I-R injury, the iOPN protein level was significantly and rapidly enhanced only in the right cortex.

There are several studies demonstrating differences between the two hemispheres in various settings including stroke. Studies by Robinson [31], [32] found spontaneous hyperactivity and a decrease in catecholamine concentrations in rats following right middle cerebral artery ligation, but not with left middle cerebral artery ligation. In these studies, right and left hemispheric infarctions were comparable in their locations and extent of tissue damage. Similarly, rat spinal fluid monoamine metabolite concentrations were reduced only on the right side compared to controls, and not on the left side [33]. The right-specific laterality has also been demonstrated in rat studies of stress-related processes where medial prefrontal cortex output neurons demonstrated an intrinsic right brain specialization in both neuroendocrine and autonomic activation [34]. These differences have been proposed to have a biochemical basis [35].

Although little is known about differences in gene expression between the right and left hemispheres in models of global ischemia, there is evidence for this possibility. Functional asymmetries have been reported in the rat brain over several decades [36]. Major laterality has been noted in the expression of protein molecules between the right and the left hippocampi in rats [37]. Thus there is precedent for the right and the left hemispheres responding differently to a cerebral insult and that the right cortex could be more susceptible to injury from I-R when compared to the left cortex. In rat hippocampi, laterality has been shown in the expression of the anti-inflammatory protein HSP70, with Samara et al. [37] demonstrating that the left side expresses nearly 3 times as much HSP70 as the right side under normal conditions. In our study, although examination of the pyknotic nuclei (indication of cell damage) showed no differences between the left and right CA1 regions of the hippocampi, it is possible that a similar laterality in expression of HSP70 might explain the differences in OPN expression between the left and right cortices following cerebral I-R.

Nitric oxide, a product of nitric oxide synthase activation, is an important mediator of ischemic brain injury [38]. There are three isoforms of nitric oxide synthase, namely neuronal nitric oxide synthase (nNOS), endothelial nitric oxide synthase (eNOS) and inducible nitric oxide synthase (iNOS). All three forms are upregulated following cerebral I-R [39]. In particular, nNOS and iNOS upregulation have been reported to occur within 15 min of cerebral I-R [40]. Several studies have revealed that OPN inhibits iNOS expression in various contexts [41], [42], [43]. It is conceivable that the right cortex is more sensitive to I-R, inducing greater iNOS expression as a response to the insult, where an upregulation in iOPN is a preventative measure to curtail the damaging effects of NO.

Cerebral insults initiate changes in expression of many proteins, including interleukin-1 beta (IL-1β), tumor necrosis factor alpha (TNFα), and the 70-kDa heat shock protein (HSP70). All these proteins have been shown to be expressed early after cerebral I-R: IL-1β within 15–30 minutes [44], TNFα within 1 hour [45], and HSP70 also within 1 hour [46]. IL-1β and TNF-α regulate nuclear factor interleukin-6 (NF-IL6), which stimulates OPN gene transcription [47]. Another potential explanation for the observed cortical differences in iOPN expression lies in the varying abundance of existing proteins in each cortex. Here we showed that iOPN protein is rapidly upregulated in the right cortex. No significant change in iOPN abundance was observed in the left cortex. While the reason for this is not known, it is likely the result of the differential expression of other genes in the two cortices of the rat brain.

We are not aware of any studies showing hemispheric differences in cerebral blood flow resulting from the 2VO/HYP procedure or differences in the internal diameters of cerebral vasculature between the two hemispheres. Thus, our results are unlikely to be explained by bilateral differences in cerebral vascular architecture, autoregulatory capacities, or metabolic/neurogenic vasomotor specializations. We suggest instead that the hemispheric differences in iOPN expression detected in our study of I-R injury are related to biochemical differences, not to differences in lesion size or location within the hemispheres. Although iOPN is thought, in part at least, to serve an adaptor (or scaffold) function facilitating signal transduction pathways, nothing is known about how its expression is controlled [48]. While the significance of our findings has yet to be explored, they highlight the emerging importance of investigating differences in the control of gene expression in the left and right hemispheres of the brain.
